# Recent Advances in Pediatric Infectious Diseases: A Review of Diagnostic and Therapeutic Strategies

**DOI:** 10.7759/cureus.94523

**Published:** 2025-10-14

**Authors:** Krishna Vamshy J, Varshini M, Neha Rathore, Ravikiran Patil, Juhita Sinha, Aditya Bhattacharjee

**Affiliations:** 1 Department of Pediatrics, Sri Siddhartha Academy of Higher Education, Tumkur, IND; 2 Department of Pediatrics, Sri Siddhartha Medical College and Hospital, Tumkur, IND; 3 Department of Microbiology, Index Medical College, Hospital and Research Centre, Indore, IND; 4 Department of Pediatrics, Bangalore Medical College and Research Institute, Bengaluru, IND; 5 Department of Nursing, Madhya Pradesh Medical Science University, Jabalpur, IND; 6 Department of Medicine and Surgery, Amrita School of Medicine, Faridabad, IND

**Keywords:** antimicrobial resistance, diagnostics, molecular assays, pediatric infectious diseases, therapeutics

## Abstract

Pediatric infectious diseases remain the leading cause of significant morbidity and mortality globally, especially among children under the age of five. Despite advances in vaccination and antimicrobial therapy, difficulties such as delayed diagnosis, increasing antimicrobial resistance, and socioeconomic inequalities constrain effective management. This review summarizes recent progress in molecular diagnostics, such as polymerase chain reaction, multiplex assays, and next-generation sequencing, and the contribution of biomarkers and host gene expression profiling to the discrimination between bacterial and viral infections to inform antibiotic stewardship. New therapeutic principles, such as immunomodulatory drugs and personalized medicine strategies, such as pharmacogenomics and therapeutic drug monitoring, are promising to enhance outcomes. Progress in pediatric vaccine antigens and schedules has further minimized the disease burden across the world. Moreover, digital health technologies and telemedicine are revolutionizing access and management of pediatric infections. However, access continues to be restricted by limited pediatric-specific clinical trials, costs, and issues of infrastructure. This integrated synthesis bridges a major knowledge gap by combining diagnostic and therapeutic innovations specific to children, providing insights to maximize clinical practice and guide future studies to promote child health globally.

## Introduction and background

In children, the burden of infectious diseases remains high concerning morbidity and mortality, even though most of these diseases have a vaccine or can be cured [1]. Despite the great progress that has been witnessed in the enhancement of childhood survival over the past few decades, infectious diseases are estimated to kill 1.5 million children under the age of five years [2]. Besides death, pediatric infections may also result in long-term complications, such as growth failure, neurodevelopmental lag, and chronic illness, which impose a long-term burden on the children who contract them and the healthcare system [3]. The immature/maturing immune system, physiological peculiarities, and patterns of exposure make children particularly vulnerable to a broad spectrum of infectious diseases, both common, such as respiratory tract infections and diarrheal diseases, and serious, such as meningitis and sepsis [4].

In the treatment of infectious diseases in children, timely and appropriate diagnosis plays a crucial role. However, it is still hard to diagnose due to an often atypical clinical presentation, overlap of symptoms with non-infectious diseases, and limitations of conventional laboratory tests that are time-consuming or lack sensitivity and specificity [5]. These diagnostic delays commonly lead to empirical antimicrobial use, which is the primary cause of the rising antimicrobial resistance (AMR) globally, a phenomenon that jeopardizes the efficacy of standard treatments [6]. This is also compounded by the fact that there are few antimicrobial agents and formulations that are specifically pediatric, and many treatments are extrapolated from the adult literature, which is unlikely to reflect pediatric pharmacodynamics, dosing, or safety profiles [7].

These problems have promising solutions in the form of new technology and therapy. Molecular diagnostic tests such as polymerase chain reaction (PCR) and multiplex assays enable high sensitivity and rapid turnaround time detection of pathogens in clinical specimens, substantially reducing the time to diagnosis [8]. Next-generation sequencing approaches provide a comprehensive identification and characterization of the pathogen, including the determination of resistance genes. Point-of-care testing devices currently in use are getting smaller and less expensive, implying that diagnosis at the bedside can be performed even in resource-constrained settings to support the clinical decision-making process and patient outcomes [9]. New host biomarkers and gene expression profiling approaches also hold the potential to differentiate bacterial and viral infections and define the severity of the disease process to allow more rational therapy.

The invention of these is a direct response to the diagnosis delays and therapy limitations that have hitherto hampered effective treatment of pediatric infections. The development of new antimicrobial agents active against the resistant pediatric pathogens and new antiviral agents specific to the infection is a significant advance in the therapeutic arena [6]. Moreover, recent technological developments of vaccines, including novel pediatric formulations and prolonged immunization schedules (e.g., conjugate vaccines or mRNA-based platforms), have also played a significant role in prevention efforts globally [10]. Pharmacogenomics also combines with therapeutic drug monitoring to facilitate personalized medicine practices, which maximize the effectiveness of drugs and minimize the adverse drug effects in children.

**Figure 1:**
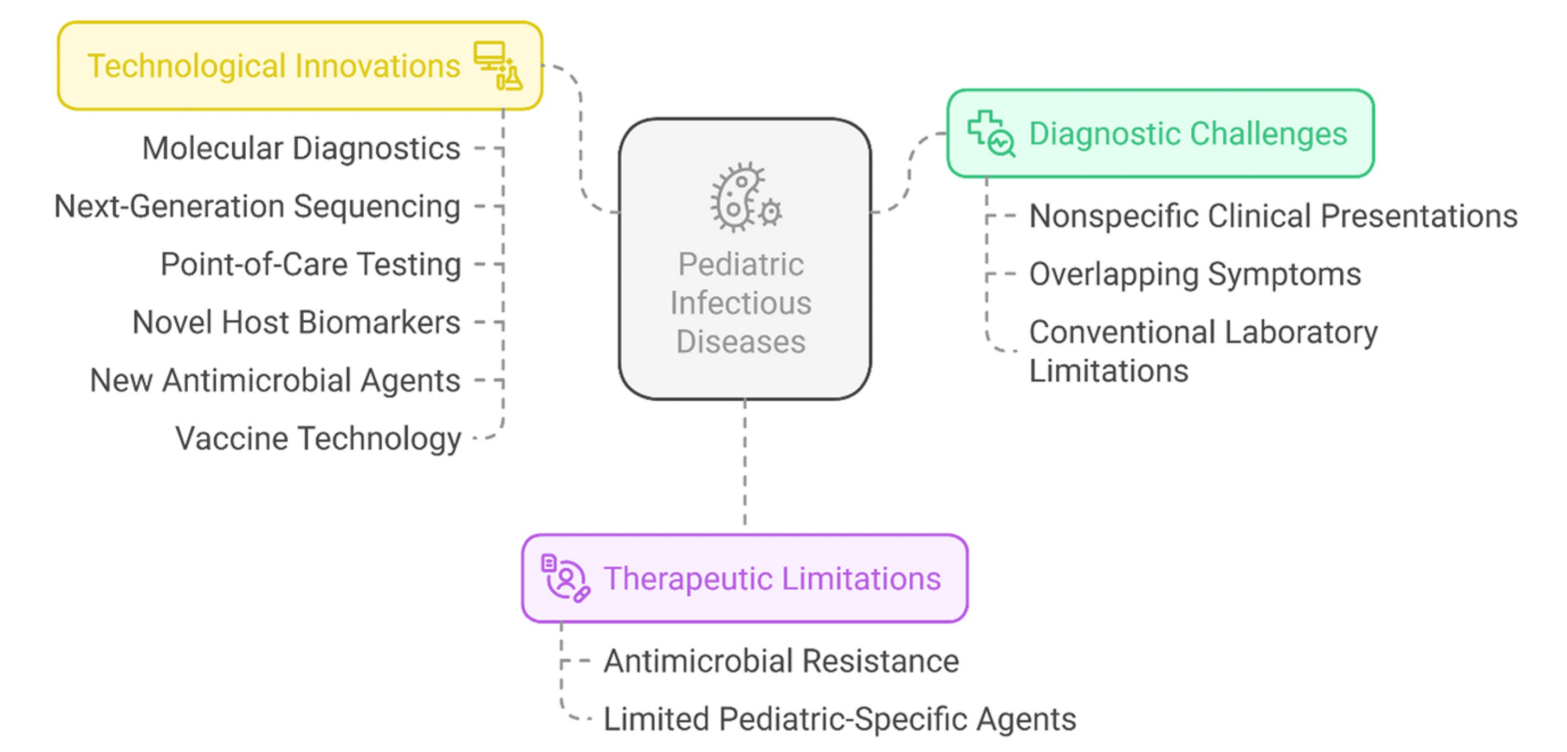
Challenges and innovations in pediatric infectious diseases. Created by authors using Napkin AI software.

Against this backdrop of promising developments, few reviews integrate diagnostics, therapeutics, vaccines, and digital health specifically for pediatrics; we aim to synthesize these domains. Existing reviews either focus on adults or describe the diagnostic and therapeutic advances in separate contexts, leaving a significant knowledge gap that may impede pediatric clinical decision-making and research. Addressing this gap is a priority to inform evidence-based clinical practice and policy. Figure 1 illustrates a summary of the key diagnostic and therapeutic challenges and recent technological solutions that can contribute to controlling pediatric infectious diseases more effectively. It isolates molecular diagnostics, antimicrobial resistance, and the development of vaccines.

Objectives of the review

This review aims to examine recent advances in diagnostic tools and methods applicable to pediatric infectious diseases, evaluate emerging therapeutic strategies and their clinical efficacy in children, and discuss the implications of these advances for clinical practice and future research directions. By providing a detailed overview of current state-of-the-art diagnostics and therapies, this review seeks to highlight opportunities to improve the management of pediatric infections and ultimately enhance child health outcomes globally.

Scope of the review

This narrative review synthesizes peer-reviewed studies published between 2008 and 2025 that evaluate recent advances in diagnostics, therapeutics, vaccination strategies, and digital health applications in pediatric infectious diseases. We included original research (randomized or observational) and systematic reviews involving patients from birth to 18 years of age, identified through PubMed, Embase, and Scopus searches. Studies were excluded if they focused exclusively on adults, were not published in the English language, or were conference abstracts without full text. By outlining these boundaries, the review clarifies its evidence base and highlights the specific knowledge gap, namely, the lack of integrated evidence on pediatric-specific diagnostic and therapeutic innovations that this review seeks to address.

## Review

Methodology

**Figure 2:**
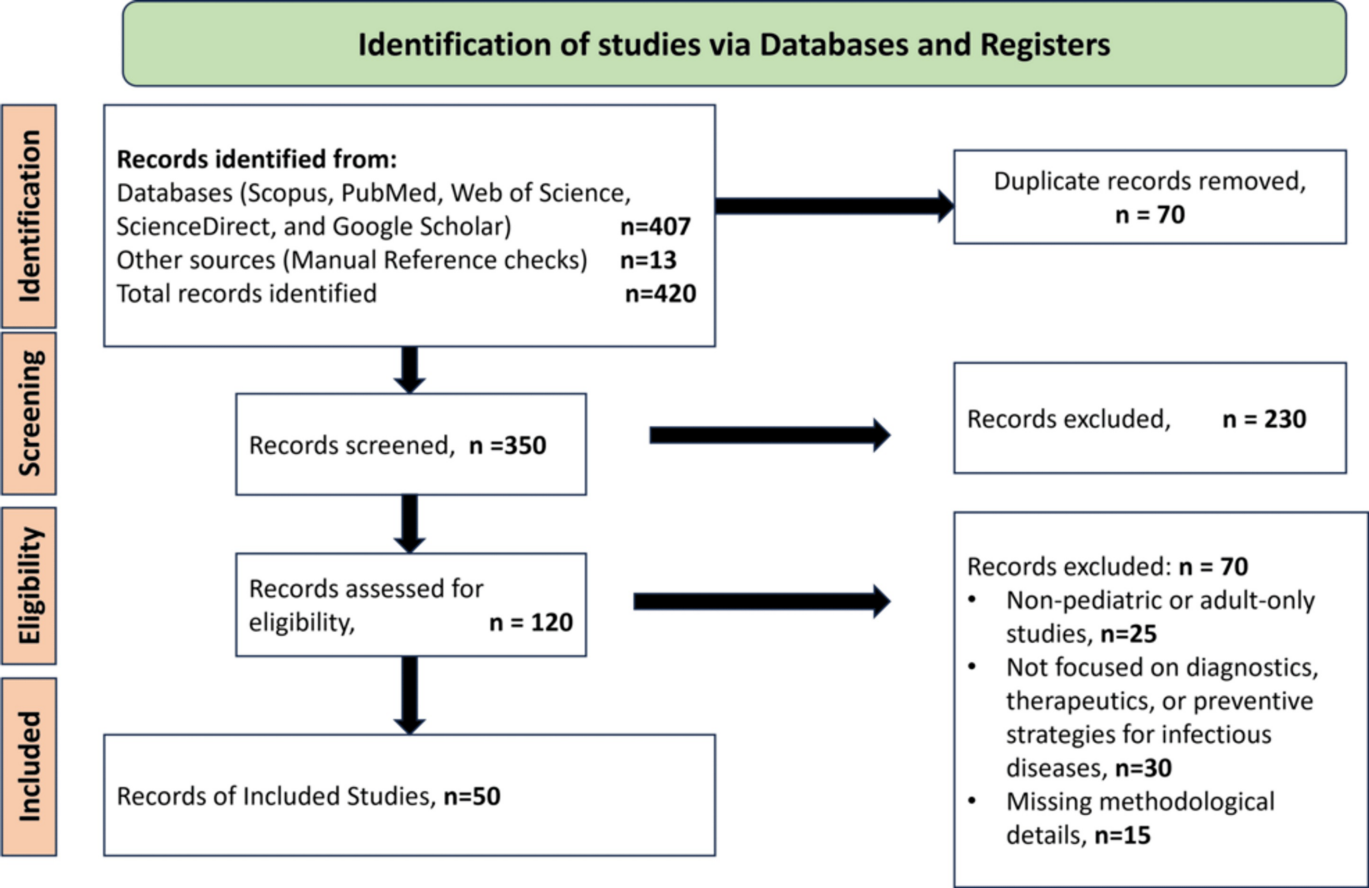
Preferred Reporting Items for Systematic reviews and Meta-Analyses (PRISMA) 2020 flow diagram illustrating study selection.

This review followed the Preferred Reporting Items for Systematic reviews and Meta-Analyses (PRISMA) 2020 guidelines. We searched PubMed, Embase, and Scopus for studies published between 2008 and 2025 on diagnostic, therapeutic, and preventive strategies for pediatric infectious diseases. Manual reference checks were also performed. After removing duplicates, all titles and abstracts were screened, and potentially relevant full texts were assessed for eligibility. Exclusion criteria included adult-only populations, non-infectious disease topics, non-English language, and conference abstracts without full text. The study identification and selection process is shown in Figure 2, which details the number of records retrieved, screened, excluded (with reasons), and the final 50 studies included in this review.

A comprehensive literature search was performed in PubMed, Embase, and Scopus for studies published between 2008 and 2025, using the following exact search strings: PubMed: (“pediatric infectious diseases”[MeSH] OR “childhood infection*” OR “pediatric infection*”) AND (“diagnostic” OR “therapeutic” OR “vaccine” OR “digital health”); Embase: (‘pediatric infectious disease’/exp OR ‘childhood infection’:ab,ti) AND (‘diagnostic’ OR ‘therapeutic’ OR ‘vaccine’ OR ‘digital health’):ab,ti AND (2008-2025)/py; Scopus: TITLE-ABS-KEY (“pediatric infectious disease*” OR “childhood infection*”) AND TITLE-ABS-KEY (“diagnostic” OR “therapeutic” OR “vaccine” OR “digital health”) AND PUBYEAR > 2007 AND PUBYEAR < 2026. Manual reference checks of included articles were also performed. As this review is a narrative review and not a systematic review or meta-analysis, registration with PROSPERO was neither required nor applicable.

This review is a narrative synthesis of heterogeneous study designs and outcomes; therefore, no meta-analysis, meta-regression, or formal GRADE assessment was performed, and no pooled statistical estimates or confidence intervals were calculated. All quantitative data are presented exactly as reported in the original studies.

Epidemiology of pediatric infectious diseases

Infectious diseases continue to be a major cause of morbidity and mortality among children globally, with a high impact on neonates and children below the age of five. Notwithstanding major global progress in vaccination, sanitation, and healthcare delivery, pediatric infectious disease still takes an extremely high toll, particularly in low- and middle-income countries where the resources are limited and health infrastructure is weak. Estimates indicate that infectious diseases cause about 15-20% of total mortality in children aged under five, which is equivalent to almost 1.5 million deaths per year [11,12]. Lower respiratory tract infections, diarrheal diseases, malaria, tuberculosis, and vaccine-preventable diseases such as measles and pertussis continue to be major causes of morbidity and mortality in children [13]. Beyond mortality, these infections cause substantial hospitalizations and long-term sequelae, including neurological impairment and growth retardation, which exert profound socioeconomic impacts on families and healthcare systems worldwide [14].

Recent Trends

Over the past two decades, global mortality rates from pediatric infectious diseases have declined owing to expanded vaccination programs and improved antibiotic access. Interestingly, the mass roll-out of pneumococcal and *Haemophilus influenzae* type b (Hib) conjugate vaccines has considerably reduced severe bacterial disease in most nations [15]. Nevertheless, new viral infections such as the respiratory syncytial virus (RSV) and new coronaviruses have assumed greater importance as leading causes of morbidity in children worldwide [16]. Adding to these challenges, AMR is compromising infection control efforts, with resistant strains of widely prevalent organisms such as *Escherichia coli*, *Klebsiella pneumoniae*, and *Staphylococcus aureus* driving treatment failures and prolonged hospitalization [17]. The emergence of multidrug-resistant tuberculosis (MDR-TB) and drug-resistant malaria adds to the epidemiological complexity [18].

Socioeconomic and Geographic Factors

The epidemiology of pediatric infectious diseases is profoundly influenced by socioeconomic and geographic determinants. Poor children are exposed to increased vulnerability in the form of malnutrition, poor sanitation, poor access to clean water, overcrowding, and limited availability of healthcare [19]. They increase susceptibility along with delaying treatment and diagnosis, thus making the transmission continuous within communities. Diarrheal diseases and pneumonia, for instance, continue to be major causes of child mortality in sub-Saharan Africa and South Asia, two poverty-stricken regions with weak healthcare systems [20]. Pathogen distribution is also affected by geographical variations; vector-borne pathogens such as malaria and dengue dominate the tropical and subtropical areas, while vaccine-preventable diseases are common in those with poor immunization coverage [11].

Urbanization and climate change have been other determinants that are changing the epidemiology of childhood infections. Urbanization tends to result in dense living conditions and low sanitation levels, posing increased risks of outbreaks. Climate change influences vector habitats as well as the dynamics of pathogen transmission, having the potential to alter the seasonal and geographic distribution of diseases like malaria, dengue, and arboviruses [12]. For example, warmer temperatures and changed rainfall patterns have recently been associated with rising malaria infection rates in new areas. Such complex interactions support the need for region-based surveillance and specific intervention measures to manage pediatric infectious diseases effectively.

Lastly, attention must be paid to the indirect effect of the COVID-19 pandemic on pediatric infectious diseases. Public health interventions such as physical distancing and enhanced hygiene lowered rates of various common infections temporarily but also interrupted regular vaccination programs, potentially raising future susceptibility to preventable diseases. Ongoing tracking will be required to grasp these dynamic changes.

Improvements in diagnostic microbiology

Early and precise diagnosis of pediatric infectious diseases is vital to directing effective treatment and enhancing outcomes. Traditional methods, such as culture and microscopy, though still a cornerstone, are frequently hampered by such issues as low sensitivity, delayed turnaround times, and inability to detect fastidious or non-culturable agents. Advances in technology have transformed diagnostic microbiology, allowing quicker, more sensitive, and multiplexed detection of pathogens to enable timely and focused therapeutic interventions.

Molecular Diagnostics (PCR and Multiplex Assays)

PCR technology has emerged as a pillar of contemporary infectious disease diagnosis. Through the amplification of target DNA or RNA sequences, PCR facilitates the rapid, highly sensitive, and specific detection of bacterial, viral, and fungal pathogens from clinical samples. In pediatric patients, PCR has revolutionized the diagnosis of diseases such as meningitis, pneumonia, and sepsis, where an early detection of pathogens is essential for timely intervention [13,14].

Multiplex PCR tests expand this functionality further by simultaneously detecting multiple pathogens within one test. Multiplex respiratory panels can detect prevalent viral and bacterial pathogens causing respiratory infections and may aid clinicians in differentiating between viral and bacterial causes, which could, in theory, reduce unnecessary antibiotic use [15,16]. However, a Cochrane review of five paediatric randomized controlled trials found that use of multiplex respiratory panels did not reduce antibiotic prescribing compared with standard care [11]. Likewise, multiplex gastrointestinal panels allow for rapid detection of enteric pathogens through stool samples, enabling quicker decisions regarding treatment of diarrheal disease in children [17].

**Figure 3:**
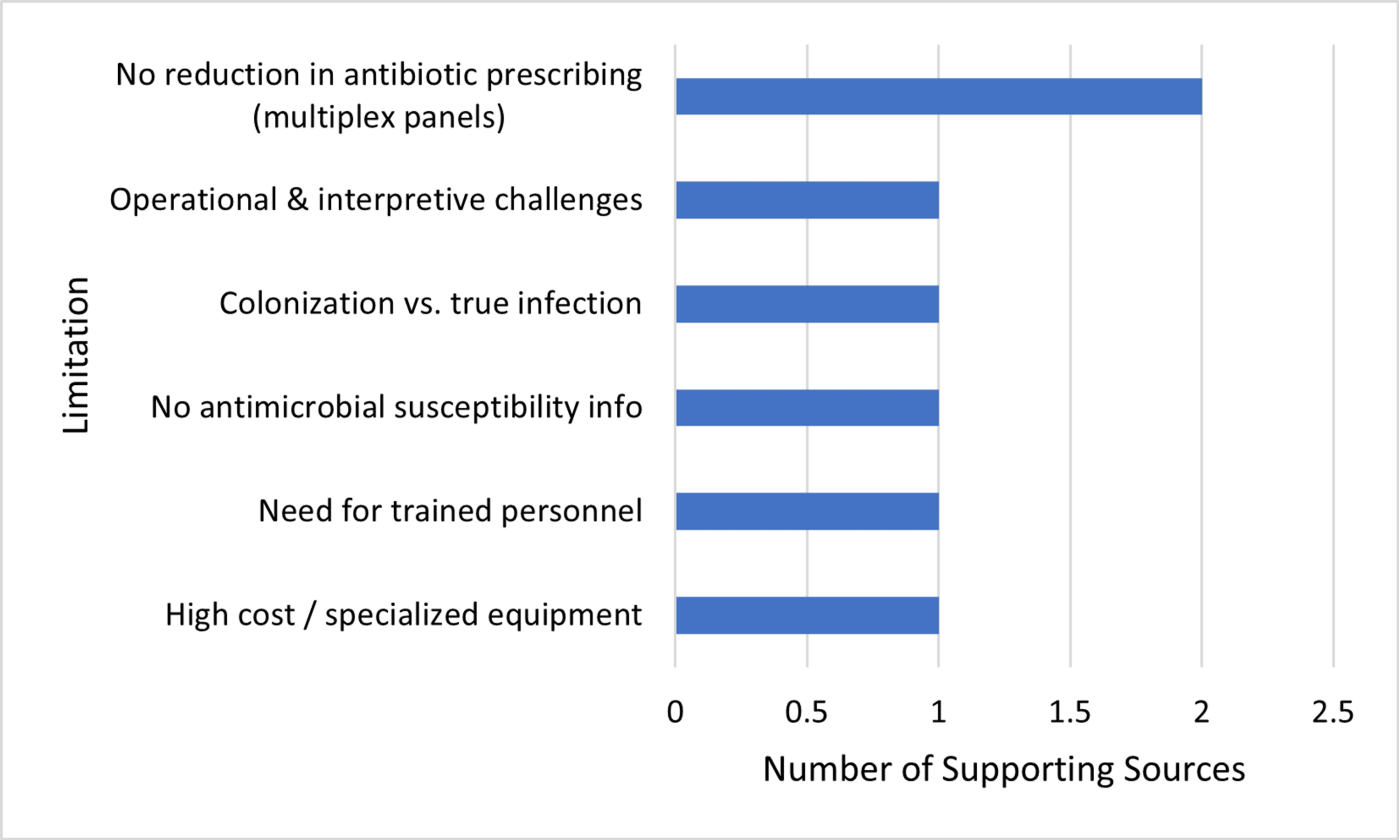
Evidence-based limitations of polymerase chain reaction (PCR) diagnostics in pediatric infectious diseases. This figure has been created by the authors based solely on the analysis of the references cited within this article.

Despite the benefits of molecular assays, there are limitations. They may be expensive, need specialized laboratory instrumentation and skilled staff, and generally do not yield antimicrobial susceptibility information, which is needed to inform therapy [18]. In addition, in some cases, PCR will identify colonizing organisms instead of legitimate pathogens, leading to overtherapy. The clinician needs to be aware of these limitations when interpreting test results. Figure 3 is divided into the key barriers restricting the application of PCR diagnostics in pediatric infectious disease care, highlighting the many-layered challenges from the economic and technical to operational and interpretive issues.

Next-Generation Sequencing for Pathogen Identification

Next-generation sequencing (NGS) technologies are a paradigm shift in infectious disease diagnosis, with the ability to perform complete, non-biased detection of all nucleic acids in a clinical sample. Metagenomic sequencing enables the detection of uncommon, new, or unusual pathogens and gives insights into the wider microbial community (microbiome) that can impact disease pathogenesis [19].

In pediatric medicine, NGS has been promising for the diagnosis of encephalitis, sepsis of unknown cause, and sophisticated infections in cases where standard diagnostics have been unsuccessful [11,20]. Sequencing is also useful for detecting AMR genes, providing useful information to inform therapy. However, limitations such as high expense, advanced data analysis, increased turnaround times, and interpretive challenges restrict its use in the general population at present [12]. As these limitations are overcome, NGS will be an important tool in the diagnosis of pediatric infectious diseases.

Rapid Antigen and Antibody Testing

Rapid antigen detection tests (RADTs) and point-of-care serology tests have become popular due to their ease of use, low expense, and rapid turnaround. RADTs for pathogens such as group A *Streptococcus*, influenza, and RSV allow for early diagnosis in outpatient and emergency facilities, allowing timely treatment and infection control interventions [13]. Serological tests for detecting pathogen-specific antibodies are useful in the diagnosis of infections with typical immunological patterns or if pathogen detection is challenging. Although these tests enhance access to diagnosis, sensitivity and specificity are very disparate. False negatives occur with some RADTs, which calls for confirmatory testing in symptomatic or high-risk patients. However, these quick tests are still essential elements of the diagnostic armory, especially in resource-poor settings where laboratory facilities are lacking [14].

Point-of-care testing in pediatrics

Point-of-care testing (POCT) refers to diagnostic tests performed at or near the site of patient care, providing immediate results to inform clinical decisions. POCT has transformed pediatric infectious disease management by overcoming barriers inherent to centralized laboratory testing, such as delays in reporting, limited access in remote areas, and infrastructure constraints.

Portable Diagnostic Devices and Bedside Testing

New technologies in microfluidics, biosensors, and miniaturized devices have made it possible to devise small, user-friendly diagnostic devices for the detection of a variety of infectious agents in small volumes of the sample. These devices allow bedside or outpatient testing for frequent pediatric infections such as respiratory viruses, urinary tract infections, and infections in the bloodstream [15,16]. For example, nucleic acid amplification tests (NAATs) and immunochromatographic assays incorporated into point-of-care devices offer quick, reliable identification of influenza, RSV, and group A *Streptococcus* in emergency rooms and clinics. Such prompt diagnostic information facilitates appropriate initiation or withholding of antimicrobials and minimizes unnecessary antimicrobial use and hospitalizations [17,18].

Advantages in Low-Resource and Emergency Settings

POCT is especially effective in low-resource environments, where laboratory capacity is absent or insufficient. Simple devices that need minimal sample preparation, no refrigeration, and minor technical skills can be used effectively in rural health centers, disaster settings, and outbreak areas, facilitating early detection and containment of infectious diseases [19]. For instance, rapid diagnostic tests (RDTs) for malaria have greatly enhanced diagnostic sensitivity and treatment in endemic regions, contributing to a decline in childhood mortality [20].

In pediatric intensive care units in emergency situations, POCT for biomarkers such as procalcitonin (PCT) and C-reactive protein (CRP) helps clinicians to judge the severity of infection and decide on antibiotic stewardship. Acceleration of the adoption of POCT has been fueled during the COVID-19 pandemic, highlighting the importance of POCT in rapid pediatric diagnosis, isolation, and infection control [1]. Despite these advantages, POCT faces challenges including variability in test accuracy, lack of standardized quality control, and integration into clinical workflows. Ensuring device reliability, operator training, and data connectivity are vital to maximizing POCT’s benefits in pediatric infectious disease management [12].

Biomarkers and Host Response Assays

The role of biomarkers in pediatric infectious diseases has become increasingly significant, given the diagnostic challenges inherent in this population. Children may manifest with overlapping, non-specific symptoms common to bacterial, viral, and non-infectious inflammatory illnesses. Here, biomarkers are objective markers representing the host’s biological response to infection, offering useful diagnostic and prognostic information. They allow early detection of severe infection, provide differentiation between bacterial and viral causes, and inform antimicrobial stewardship, important in reducing inappropriate use of antibiotics and mitigating antimicrobial resistance. Table 1 summarizes commonly used biomarkers in the diagnosis and management of pediatric infectious diseases.

**Table 1:** Common biomarkers in pediatric infectious diseases: types, uses, accuracy, and limitations.

Biomarker	Type	Clinical use	Sensitivity and specificity	Limitations	References
Procalcitonin (PCT)	Peptide precursor	Differentiates bacterial vs. viral infections; guides antibiotic therapy; monitors treatment response	High sensitivity and specificity for bacterial infections (70–90%)	Elevated in non-bacterial inflammation (e.g., trauma); variable cut-offs in neonates	[13]
C-reactive protein (CRP)	Acute phase protein	Assesses inflammation; supports diagnosis and treatment monitoring	Moderate sensitivity and specificity; rises more slowly than PCT	Nonspecific; elevated in any inflammatory state; delayed peak limits early diagnosis	[6]
White blood cell (WBC) count	Hematologic marker	General infection and inflammation indicator	Variable, limited specificity	Influenced by many factors, poor discrimination between infection types	[17]
Interleukin-6 (IL-6)	Cytokine	Early marker of inflammation and sepsis	High sensitivity in early sepsis detection	Limited availability; rapid changes complicate interpretation	[9]
Proadrenomedullin (ProADM)	Peptide biomarker	Prognostic marker for severity and outcomes in sepsis and pneumonia	Promising sensitivity and specificity	Requires further validation in pediatrics	[10]
Host gene expression profiles	Molecular signatures	Differentiates bacterial vs. viral infections; predicts disease severity	High accuracy (>90%) in research settings	Expensive, complex, not yet widely available clinically	[5]

Procalcitonin and C-Reactive Protein

PCT, a pro-hormone of calcitonin, is primarily synthesized in response to bacterial infection, with concentrations increasing sharply within two to four hours of the onset of infection and being directly related to the severity of the infection [21]. In children, PCT is more specific and sensitive than conventional markers such as white blood cell counts, especially in diagnosing bacterial sepsis and meningitis over viral infections [22]. Several studies have demonstrated that antibiotic exposure can safely be decreased by PCT-guided antibiotic therapy, notably in respiratory infections and febrile infants, without affecting clinical outcomes [23,24]. The largest paediatric data set to date, an individual-patient meta-analysis of 2,024 children [12], confirmed that PCT-guided therapy significantly reduced antibiotic duration without compromising safety. Its value, however, depends on clinical situation and patient population, and interpretation should take age-related reference ranges and comorbidities into account [25]. Clinical trials using PCT algorithms have demonstrated reductions of as much as 30% in the duration of antibiotics for hospital-acquired lower respiratory tract infections in children, highlighting its value for stewardship programs [26].

CRP is an acute-phase reactant that is synthesized by hepatocytes in response to inflammation. While not as specific as PCT, its availability and cost make it a useful tool globally [27]. CRP increases more gradually than PCT, usually 24-48 hours after the onset of infection, making its initial diagnosis more restricted but valuable for detecting disease progression or remission [28]. Serial measurement of CRP is a complement to clinical acumen, especially with bacterial infections, including pneumonia, osteomyelitis, and urinary tract infection [29].

Each of these biomarkers has limitations. The values can be elevated in non-infectious inflammatory states, such as autoimmune conditions, trauma, or postoperative situations, risking confusion with clinical interpretation [30]. Cut-off values also vary across the different pediatric age groups and must be applied with careful judgment. The optimum practice is still combining biomarker information with clinical evaluation and other diagnostic tests.

Emerging Host Gene Expression Profiling

Expanding on classical biomarkers, host gene expression profiling investigates signatures of immune-associated gene activation, providing a more specific method for diagnosing infection. Differential mRNA expression signatures induced by bacterial versus viral infection are identified using these assays, usually proving more accurate than individual protein markers [12]. Transcriptomic signatures of more than 90% accuracy in the classification of febrile illness have been shown in pediatric groups, with the potential to revolutionize antibiotic prescribing through the avoidance of unnecessary use [23]. For example, comparing two-transcript RNA signatures (FAM89A and IFI44L) has consistently differentiated bacterial from viral infections in the setting of an emergency department [4].

Outside of diagnosis, host gene expression signatures can forecast the severity of disease by detecting hyperinflammatory or immunosuppressive conditions, which will assist in risk stratification and individualized treatment strategies [7]. Widespread clinical application is challenging for their promise due to prohibitive costs, technical requirements, and the necessity of rapid turnaround times conducive to acute care environments. Continued research aims to simplify these assays and make them available for POCT, much to the benefit of pediatric infectious disease care, particularly in low-resource settings [16].

Antimicrobial resistance in pathogens in children

AMR is an emerging worldwide threat, majorly complicating the treatment of infections in children. Resistant pathogens cause more morbidity, mortality, prolonged hospital stays, and increased healthcare expenditures. Knowledge of prevailing resistance patterns and the clinical implications is important for the rationalization of treatment strategies.

Current Status of Resistance Patterns

**Figure 4:**
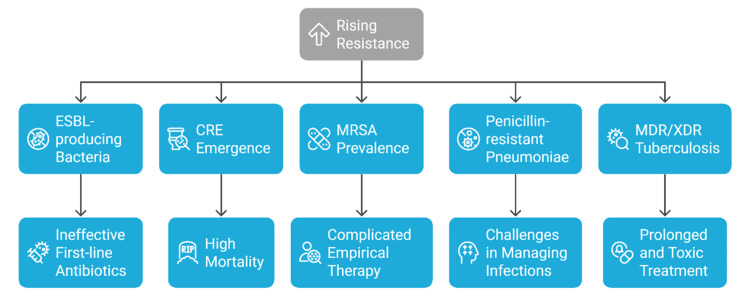
Key antimicrobial resistance patterns impacting pediatric infectious diseases. Created by authors using Napkin AI software.

Resistance in the major child pathogens is increasing globally. *Escherichia coli* and *Klebsiella pneumoniae*, leading to neonatal sepsis and urinary tract infections, increasingly produce extended-spectrum beta-lactamases (ESBLs), making most first-line drugs ineffective [17,28]. Carbapenem-resistant *Enterobacteriaceae* (CRE) have been reported in pediatric intensive care units, with a recent cohort study of bloodstream infections (2018-2022) showing that 52% (114/219) of Gram-negative bloodstream infection episodes in hospitalized children were due to CRE, with a median patient age of 11 months and nearly half having prior CRE colonization or infection [29]. Methicillin-resistant *Staphylococcus aureus* (MRSA) remains a common cause of skin, soft tissue, bacteremia, and pulmonary infections in children, both in communities and hospitals [30]. Clindamycin and macrolide resistance make empirical treatment complicated. Additionally, penicillin-resistant *Streptococcus pneumoniae* becomes problematic in the treatment of pediatric pneumonia and meningitis worldwide [31]. In childhood tuberculosis, multidrug-resistant (MDR) and extensively drug-resistant (XDR) strains have appeared, especially in high-burden nations, requiring longer and more toxic treatment courses, and contributing to increased morbidity [22]. Figure 4 illustrates the growing frequency of AMR among pediatric pathogens and its consequences on treatment in terms of ineffective first-line antibacterials, high mortality, complex empirical treatment, difficult management, and long-term toxic treatments.

Implications for Therapy Choice

The escalation of AMR demands judicious antibiotic prescribing informed by local surveillance data and antimicrobial susceptibility testing. Empirical treatment regimens require frequent updating to reflect current resistance patterns [12]. Although broad-spectrum antibiotics may be necessary for severe infections, their use should be carefully limited and de-escalated based on culture results to reduce selection pressure [5].

Rapid diagnostics and biomarker-guided therapy support timely narrowing or cessation of antibiotics, reducing unnecessary exposure and resistance development [23]. Antimicrobial stewardship programs designed for pediatric populations are vital in promoting appropriate use, optimizing dosing, and improving clinical outcomes [30]. Additionally, investment in global surveillance and reporting networks enables real-time tracking of resistance trends, informing both clinical practice and public health policies.

Novel antimicrobial agents and therapeutics

The development of new antimicrobial agents and adjunctive therapies is crucial to address resistant pediatric infections and improve patient outcomes. Recent advances encompass novel antibiotics, antiviral drugs, and immunomodulatory therapies.

New Antibiotics and Antiviral Drugs Approved or in the Pipeline

Several new antibiotics with activity against MDR Gram-negative bacteria have been approved or are in advanced clinical trials for pediatric use. Agents such as ceftazidime-avibactam, meropenem-vaborbactam, and cefiderocol exhibit efficacy against resistant pathogens causing complicated urinary tract infections, pneumonia, and bloodstream infections [8,14]. Pharmacokinetic and safety studies in children have informed dosing recommendations, though long-term data remain limited. Novel oral antibiotics under development aim to improve outpatient management and reduce hospital stays, including siderophore cephalosporins that exploit bacterial iron uptake mechanisms to enhance activity [2]. These agents offer hope for more effective and convenient therapies for resistant infections.

On the antiviral front, palivizumab, a monoclonal antibody against RSV, is well established for prophylaxis in high-risk infants [26]. Newer small-molecule antivirals and long-acting monoclonal antibodies targeting RSV and influenza are in clinical development, promising enhanced prevention and treatment options. The COVID-19 pandemic accelerated the introduction of antivirals such as remdesivir and molnupiravir for pediatric SARS-CoV-2 infection, demonstrating rapid innovation in response to urgent clinical needs [10]. Table 2 summarizes recently approved and investigational antimicrobial agents and antiviral drugs relevant to pediatric infectious diseases.

**Table 2:** Novel antimicrobial agents and antiviral drugs in pediatric infectious diseases.

Drug name	Target pathogen(s)	Type	Pediatric approval status	Current clinical trial phase/Notes	References
Ceftazidime-avibactam	Multidrug-resistant Gram-negative bacteria (e.g., extended-spectrum beta-lactamase, carbapenem-resistant *Enterobacterales*)	Antibiotic	Approved for pediatric use	Phase IV post-marketing surveillance is ongoing	[8]
Meropenem-vaborbactam	Carbapenem-resistant *Enterobacteriaceae*	Antibiotic	Limited pediatric data, expanded studies underway	Phase III pediatric PK and safety studies	[27]
Cefiderocol	Carbapenem-resistant Gram-negative bacteria	Antibiotic	Pediatric trials ongoing	Phase II/III pediatric clinical trials	[8,19]
Siderophore cephalosporins (e.g., cefiderocol)	Multidrug-resistant Gram-negatives	Antibiotic	Investigational	Early pediatric trials	[9]
Palivizumab	Respiratory syncytial virus (RSV)	Monoclonal antibody	Approved for high-risk infants	Widely used, long-acting antibodies in development	[10]
Remdesivir	SARS-CoV-2 (COVID-19)	Antiviral	Emergency use authorization in pediatrics	Approved; pediatric dosing and safety studies ongoing	[21]
Molnupiravir	SARS-CoV-2	Antiviral	Emergency use authorization; limited pediatric data	Ongoing pediatric trials	[12]
New RSV small-molecule antivirals (e.g., Presatovir)	RSV	Antiviral	Investigational	Phase II/III pediatric trials	[16]
New Influenza antivirals (e.g., Baloxavir)	Influenza virus	Antiviral	Approved in adults; pediatric studies ongoing	Phase III pediatric trials	[3]

Use of Immunomodulators and Adjunct Therapies

Adjunctive immunomodulatory treatment is being explored for its possible role in modulating deleterious host inflammatory responses during infection. Corticosteroids have been shown to decrease morbidity in bacterial meningitis and severe pneumonia by suppressing excessive inflammation [4]. Intravenous immunoglobulin (IVIG) is used in certain severe infections to confer passive immunity and regulate immune reactions [19].

New therapies aiming at specific cytokines, including interleukin-6 and tumor necrosis factor-alpha inhibitors, are being explored to minimize sepsis-induced organ damage and mortality [11]. Targeted therapy might represent the beginning of an era of personalized adjunct therapy in pediatric infections. Supportive care, such as probiotics to reestablish gut microbiota homeostasis and nutritional support to augment immune function, is being researched, especially in neonates and immunocompromised patients [28]. These adjuncts can lower infection risk and enhance recovery, but need more rigorous testing.

Vaccination Strategies and Developments

Vaccination is among the most effective public health strategies for decreasing infectious diseases in children worldwide. Pediatric immunization programs over the last few decades have significantly reduced the morbidity and mortality from many infections. Recent vaccine advancements have been aimed at improving immunogenicity, safety, and access, as well as against emerging variants of pathogens and existing logistical issues. Updates on Pediatric Vaccines: New Formulations and Schedules New pediatric vaccine development has recently focused on extending pathogen coverage, enhancing formulations, and fine-tuning immunization schedules to enhance uptake and efficacy. For example, pneumococcal conjugate vaccines (PCVs) have progressed from the seven-valent (PCV7) to 10- and 13-valent formulations and effectively lowered invasive pneumococcal disease and related complications in vaccinated children globally [3]. Candidate next-generation PCVs currently in clinical trials target more than 20 serotypes, potentially extending protection against upcoming strains resistant to current vaccines.

Likewise, meningococcal vaccines have improved with conjugate formulations against serogroups A, C, W, and Y, and protein-based vaccines against serogroup B, which collectively provide broad protection against invasive meningococcal disease [15]. These vaccines have shown considerable efficacy in decreasing the incidence of disease, particularly in epidemic-prone areas. Moreover, combination vaccines such as the hexavalent formulation (protecting against diphtheria, tetanus, pertussis, polio, Haemophilus influenzae type b, and hepatitis B) rationalize immunization schedules by limiting the number of injections and healthcare visits, enhancing compliance and vaccination rates [29]. Continued research is underway regarding flexible dosing intervals and fractional dosing approaches to optimize immunization schedules according to epidemiological settings and enhance global vaccine coverage. The development of new vaccine platforms, such as mRNA and viral vector technology, has transformed vaccine development, as seen in the swift rollout of COVID-19 vaccines [17]. Outside of SARS-CoV-2, these platforms have the potential to rapidly adapt to new pediatric pathogens and could potentially facilitate the development of vaccines against previously recalcitrant infections such as RSV and cytomegalovirus. Pediatric trials are imminent, with the promise of future extension of these new vaccine methods. Table 3 compiles major pediatric vaccines, including their formulation, target age, efficacy, and current coverage status, showing recent developments and future directions in pediatric immunization.

**Table 3:** Summary of key pediatric vaccines: formulations, target ages, and coverage.

Vaccine	Type/Valency	Target age group	Efficacy	Coverage/Notes	References
Pneumococcal conjugate vaccine	PCV10, PCV13, Next-gen (20+)	Infants, toddlers	High efficacy against invasive disease	Widely implemented, next-gen vaccines in trials	[21,32]
Meningococcal vaccine	Conjugate (A, C, W, Y), Protein B	Infants, adolescents	Effective in outbreak control and prevention	Targeted use in high-risk/epidemic areas	[15]
Hexavalent vaccine	Combination (DTP, Polio, Hib, HBV)	Infants	High immunogenicity; reduces injection burden	Standard in many national programs	[23]
COVID-19 mRNA vaccine	mRNA platform	Children 6 months and older	Strong immunogenicity; rapid adaptability	Pediatric trials are ongoing globally	[24]
Rotavirus vaccine	Live attenuated	Infants	Reduces severe gastroenteritis	Global uptake variable; recommended by the WHO	[25]

Impact on Infectious Disease Prevention

Child vaccination has produced dramatic declines in disease burden globally. The almost eradication of poliomyelitis and substantial declines in measles, rubella, diphtheria, and other vaccine-preventable infections demonstrate the striking impact of sustained immunization efforts [24]. Herd immunity from high vaccine uptake also covers susceptible unvaccinated populations, such as neonates and immunocompromised children. Vaccination not only lowers infection rates but also secondary antibiotic consumption by avoiding bacterial complications, such as pneumonia and otitis media, and hence indirectly fighting AMR [7]. However, issues such as vaccine hesitancy, disinformation, and vaccine inequities, especially in low-income nations, risk diminishing these achievements. Improvement of public health infrastructure, education, and cooperation between countries is still essential to increase vaccine coverage and maintain control of diseases. Emerging approaches, such as maternal immunization, add further protection by passing antibodies to neonates, closing the gap of vulnerability early in life until active immunization can start. Digital technologies and mobile health platforms are being used more and more to enhance vaccine delivery, monitor coverage, and combat misinformation, serving as valuable adjuncts to conventional immunization programs.

Therapeutic drug monitoring and personalized medicine

The optimization of antimicrobial therapy in the pediatric population demands cautionary consideration of the specialized pharmacokinetic and pharmacodynamic properties of children. Therapeutic drug monitoring (TDM) and the practices of personalized medicine, such as pharmacogenomics, are evolving as central strategies for tailoring treatment, optimizing efficacy, and reducing toxicity.

Customizing Dosing of Antibiotics in Pediatrics

Children display dynamic physiological alterations, influencing drug absorption, distribution, metabolism, and excretion, which require dosing adjustments by age [31]. Extrapolated dosing regimens based on adults' dosing are usually inadequate and could lead to subtherapeutic levels or toxicity in children, especially neonates and infants. TDM allows quantification of drug levels in blood or fluids and informs the adjustment of doses for the achievement of therapeutic targets. This is better established for drugs with a narrow therapeutic window and high risks of toxicity, including aminoglycosides and vancomycin [32]. For neonates and critically ill children, TDM assists with optimizing the dose based on the varying organ function and volume status, enhancing the outcome of treatment and safety. Population pharmacokinetics and Bayesian dosing models have made advances that enable the incorporation of patient-specific variables (renal function, age, weight) with observed drug levels to tailor dosing regimens [33]. These are available to make dynamic adjustments in dose and can be incorporated into clinical decision-support systems, enabling precision medicine in real-time.

Pharmacogenomics and Its Potential Role

Pharmacogenomics analyzes genetic variation to affect drug metabolism and response, holding the promise to further tailor antimicrobial treatment in children. Genetic polymorphisms in genes for cytochrome P450 enzymes, drug transporters, and drug targets can significantly influence drug pharmacokinetics and toxicities [34]. For instance, genetic polymorphisms in genes that affect aminoglycoside metabolism could make some children susceptible to ototoxicity, holding potential for genotype-directed therapy to improve safety [35]. Likewise, pharmacogenomic knowledge may streamline dosing of antivirals and other antimicrobials by predicting rates of metabolism and efficacy. Even with these promising opportunities, however, pharmacogenomics for children is in its infancy because of the scarcity of pediatric-specific data, the expense of genetic tests, and issues of consent and data privacy. Research on creating rapid, low-cost point-of-care genotyping and on validating clinical algorithms continues. Pharmacogenomics integration with TDM and electronic health records will most probably facilitate better personalized antimicrobial approaches in the future [36].

Management of Emerging and Re-emerging Pediatric Infections

SARS-CoV-2, the causative virus of COVID-19, presented unprecedented challenges to pediatric infectious disease care globally. Children seemed to be less affected in the early pandemic phase; however, later waves showed severe, unique complications in children, including pediatric MIS-C, acute respiratory distress syndrome, and long-term sequelae [37]. The sudden onset of viral mutations, some with enhanced transmissibility and immune evasion capacity, added another layer of complexity to vaccine strategy and therapeutic protocols in children. This ever-changing situation compelled healthcare systems to quickly redefine pediatric pathways of care, redirect resources, and create new treatment protocols with limited pediatric-specific evidence. RSV continues to be a major cause of lower respiratory tract disease and hospitalization in infants and young children globally, frequently necessitating intensive care [38]. Interestingly, the COVID-19 pandemic and resultant social distancing broke normal RSV spread patterns, resulting in unusual seasonality, delayed emergence, and heightened case severity in certain areas [39]. These sudden spikes taxed pediatric critical care capacity and highlighted the need for elastic surveillance and response mechanisms with the ability to respond to changing epidemiological patterns.

Other re-emerging infectious diseases such as measles, pertussis, and dengue remain a cause of high morbidity and mortality among children, especially in the low- and middle-income countries. Low vaccine coverage, usually worsened by pandemic-induced interruptions, coupled with urbanization and climate change, enables outbreaks and makes control a challenge [40,41]. Moreover, zoonotic diseases such as avian influenza and emerging enteroviruses also continue to threaten pediatric populations, highlighting the importance of strong global surveillance and early warning networks. Successful control of emerging and re-emerging childhood infections involves a multifaceted strategy. Early identification via strengthened systems of surveillance and integrated laboratory networks, equipped with the use of sophisticated molecular diagnostics and genomic sequencing, is achieved, facilitating timely pathogen identification and characterization [42]. This ability is critical to inform targeted public health interventions and clinical management. Vaccination continues to be the pillar of outbreak prevention. The process of rapid development and emergency use of pediatric COVID-19 vaccines showed that rapid pediatric vaccine responses were possible during public health emergencies [43]. Continuation and strengthening of routine childhood immunization activities are essential to avert vaccine-preventable disease resurgence during global health emergencies. IPC practices modified for pediatric healthcare facilities are essential to minimize nosocomial spread during outbreaks. Practices entail patient cohorting, strict use of personal protective equipment, cleaning of the environment, and robust staff training [44]. Strong communication with families and communities reinforces compliance with IPC guidelines and tackles misinformation, which is especially important in pediatric settings. Rapid development and dissemination of pediatric-specific clinical guidance facilitate frontline healthcare workers in embracing evidence-based practices for diagnosis, treatment, and prevention in developing outbreaks [45]. Multidisciplinary coordination between pediatricians, infectious disease experts, epidemiologists, and public health authorities maximizes clinical care and epidemic response.

Telemedicine and digital health in pediatric infectious diseases

The COVID-19 pandemic sped up the integration of telemedicine and digital health technology, revolutionizing pediatric infectious disease practice. Telehealth allows for safe and equitable assessment and management of infectious disease with reduced risk of exposure for patients, families, and medical professionals.

Role of Telehealth in Diagnosis and Follow-Up

Remote clinical examinations are made easy through telemedicine platforms, especially useful during infectious disease epidemics when reducing face-to-face interaction is critical [46]. Telehealth in pediatrics facilitates initial assessment of febrile illness, monitoring of chronic infectious disease, and follow-up, improving healthcare access for families in rural or underserved locations. Virtual triage enables clinicians to measure the severity of illness, to recognize children who need immediate in-person evaluation, and to decrease unnecessary visits to the emergency department, saving healthcare resources during surges [47]. Research shows high levels of satisfaction among providers and parents about the convenience of telehealth and its function in overcoming geographic distances [48]. Telehealth has its limitations, though. Physical examination is limited, which makes it difficult to diagnose, particularly in young children who cannot verbalize symptoms [49]. Furthermore, variations in internet connectivity, device ownership, and digital competency can restrict fair usage of telemedicine. Regulatory, reimbursement, and licensure systems are changing but remain obstacles to universal uptake.

Utilization of Artificial Intelligence and Digital Technologies in Decision Support

Digital health technologies and artificial intelligence (AI) are increasingly being incorporated into pediatric infectious disease care to support clinical decision-making. Machine learning algorithms used in electronic health records have the potential to forecast the risk of infection, recognize early warning signs of sepsis, and sense outbreaks, allowing timely intervention [50]. AI-based sepsis alert systems in pediatric intensive care units are effective in enhancing early diagnosis and decreasing mortality. Evidence-based guidelines and calculators integrated into clinical decision-support tools help clinicians differentiate between viral and bacterial infections, thereby aiding in antibiotic stewardship [30]. Mobile health applications and wearable sensors enable remote monitoring of vital signs and symptom evolution, allowing for the early detection of clinical deterioration and enabling proactive management adjustments.

**Figure 5:**
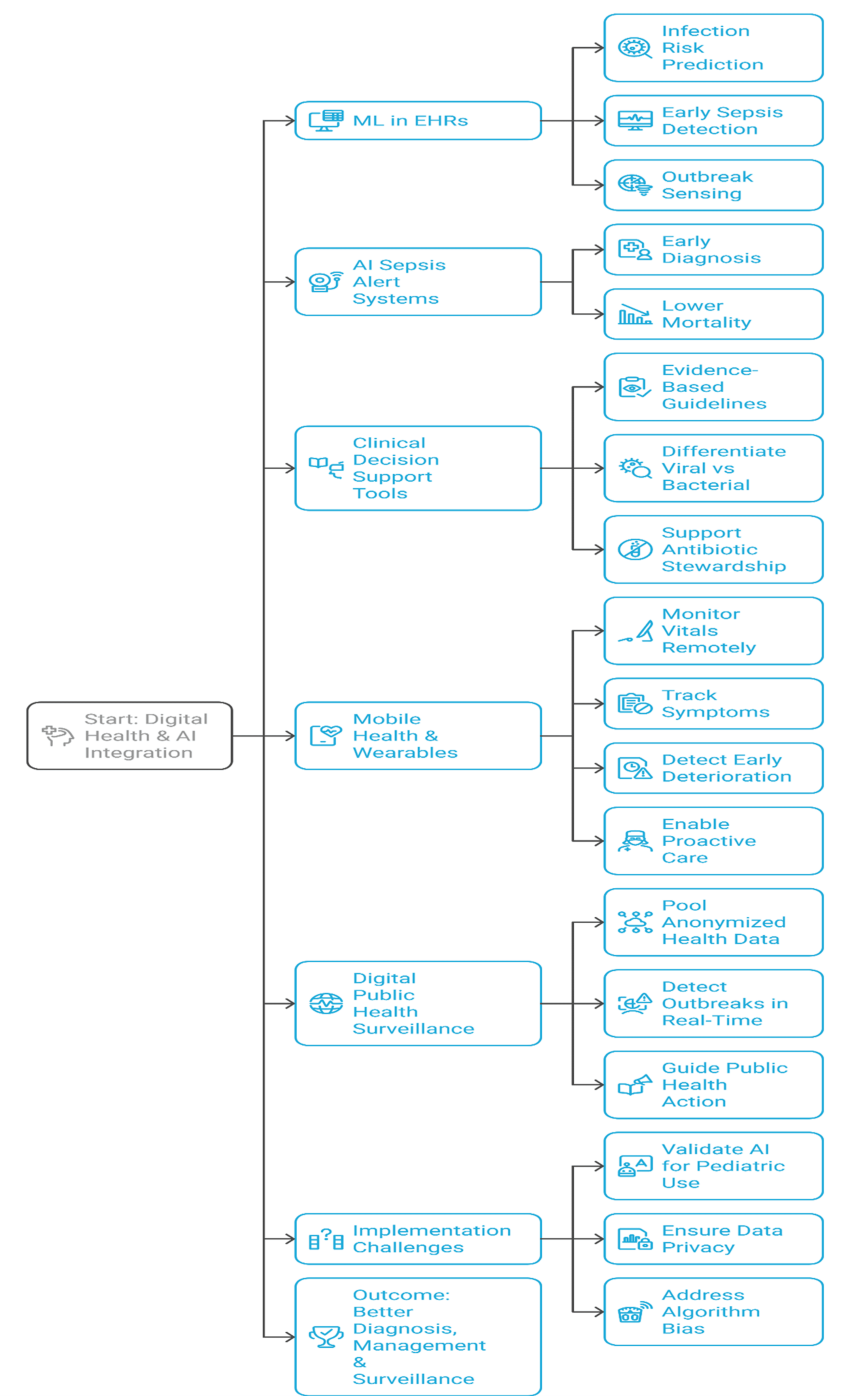
Key applications of artificial intelligence and digital technologies in pediatric infectious disease management. Created by authors using Napkin AI software.

In addition to clinical treatment, digital platforms pool anonymized health information for public health surveillance, enhancing real-time outbreak detection and informing public health responses. However, roll-out at scale depends on validation of AI algorithms in pediatric populations, data privacy protections, and reduction of biases in algorithm training data sets. Figure 5 shows how sophisticated digital health solutions facilitate early diagnosis, proactive patient management, and timely public health responses, and, ultimately, enhance outcomes and surveillance for pediatric infectious disease.

Limitations

Substantial heterogeneity within included studies presented challenges to synthesis and interpretation. Differences in study designs, patient populations, diagnostic criteria, and outcome measures precluded meta-analyses or direct comparisons. The relative lack of pediatric-specific clinical trials means many therapeutic advances are extrapolated from adult data, which may not reflect pediatric pharmacodynamics or safety profiles. Translation of research gains into practice is further hindered by prohibitive costs, sparse healthcare infrastructure, complex regulations, and variability in resource availability across regions. Ethical issues unique to pediatric populations, such as consent and long-term safety monitoring, add additional complexity to implementation.

Future recommendations

Future investment must focus on large-scale pediatric clinical trials that fill key gaps in safety, pharmacokinetics, and efficacy of new diagnostics and therapeutics, especially in neonates and diverse populations. The combination of multi-omics methods, genomics, proteomics, and metabolomics need to be coupled with AI-based diagnostic tools to drive early detection and tailored treatment approaches, requiring major investment in both research and implementation infrastructure. In addition, supportive antimicrobial stewardship programs for pediatric care must be strengthened in order to fight emerging resistance by facilitating optimal antibiotic use. Universal vaccination coverage is still a priority worldwide, together with speeding up development and equitable access to vaccines against emerging and re-emerging pediatric pathogens such as respiratory viruses and vector-borne diseases. In addition, increasing international cooperation through sharing data, harmonized standards, and mobilization of resources is crucial in eliminating infectious disease inequalities and ensuring that all children derive benefits from advances. Together, these complementary strategies will propel sustainable gains in pediatric infectious disease prevention and care globally.

## Conclusions

This review combines recently developed innovations in diagnostics, therapeutics, and digital health uniquely for clinicians, researchers, and policymakers to offer them a complete vision of existing advances and important future directions in the management of pediatric infectious diseases. Through the integration of advances in molecular and host-response diagnostics, new antimicrobial agents, personalized medicine strategies, extended vaccination regimes, and advancing telemedicine and AI solutions, it shows how these multidimensional advances together enhance the precision, pace, and efficacy of pediatric care. The concentration on pediatric-specific issues and solutions emphasizes the value of scaling interventions to fit the distinctive needs of children. Despite these encouraging developments, remarkable gaps persist, foremost of which is the lack of strong pediatric clinical trials, fair access to innovations, and successful translation of research into practice. There is a need for increased work in pediatric-specific research, faster utilization of proven technologies, and supportive health policies that counteract disparities and promote international collaboration. Adopting these focused approaches will become critical to surmounting entrenched issues of AMR, new and re-emerging pathogens, and health inequities, which will, in turn, enhance clinical outcomes and protect child health globally.
